# Supplementary material for: Interacting with Hemoglobin: Paracoccidioides spp. Recruits hsp30 on Its Cell Surface for Enhanced Ability to Use This Iron Source
Source: J Fungi (Basel). 2021 Jan 1;7(1):21. doi: 10.3390/jof7010021 (PMC7823998; doi:10.3390/jof7010021)
Supplement: Supplementary file 1 [file jof-07-00021-s001.pdf]

Supplementary Table 1. Oligonucleotide sequences for RT-qPCR used in the present study

Genes	Forward sequences (5′-3′)	Reverse sequence (5′-3′)
HSP30	ACTATGAAAGCCACCGCTCC	TCTGAGAATCTGTGAACTCGAT
GAPDH	CATGCGGGTGCCCACTGC	AGAATAGCCGAGAATGCCCTT

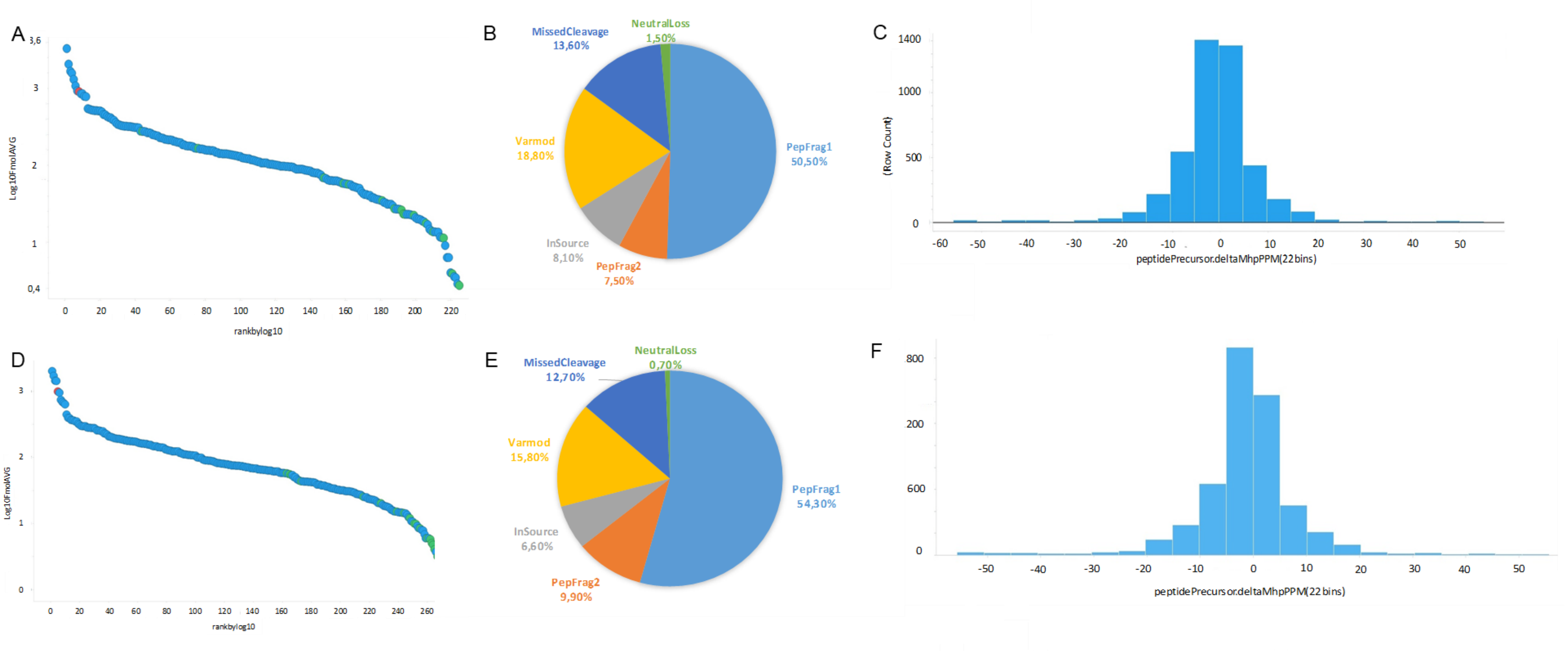

Supplementary Figure 1. Quality analysis of the obtained proteome. Quality charts of the proteomic analysis related to *P. lutzii* cell wall Fraction 1 obtained in the presence of hemoglobin (**A**) or BPS (**D**): Dynamic range of proteins detection, considering their abundance and molecular weight. Blue circle: proteins identified on a regular basis. Green circle: proteins identified in reverse. Red circle: standard (PHB). Type of peptides detection **(B)** (Hb) and (**E)** (BPS): PepFrag1 and PepFrag2: mode of identification in the *Paracoccidoides* database by PLGS. VarMod: variable modifications. InSource: fragmentation occurred at the ionization source. MissedCleavage: loss of cleavage by trypsin. NeutralLoss: loss of precursors of water, ammonia and / or phosphoric acid. Mass Accuracy (**C)** (Hb) and (**F)** (BPS): The graph shows accuracy in the detection of peptide masses, in which 61.9% of the peptides identified in haemoglobin condition and 62,2% of the peptides identified in BPS condition had a mass error of 5 ppm. Additional analysis

showed false positives in 0.63% of the detected peptides from haemoglobin condition, low value that allows proteomic analysis to be performed. False positives were not identified in the BPS condition. Analysis based on (GEROMANOS et al., 2009).

Supplementary Table 2. Upregulated proteins identified in Fraction 1 (F1) of cell wall, extracted by treatment with SDS, after *Paracoccidioides lutzii* yeast cells exposition to hemoglobin for 48 hours

**Accession Number**[a]	**Description**[b]	**Score**[c]	**Expression levels Ratio (Hb/BPS)**[d]	**SignalP**[e]	**SecretomeP**[f]
PAAG_00435	(R)-benzylsuccinyl-CoA dehydrogenase	1048,6	1.92	-	-
PAAG_00773	14-3-3 family protein epsilon	2559,6	1.75	-	-
PAAG_00871	30 kDa heat shock protein	754,8	*	-	0.786
PAAG_01870	3-oxoacyl-[acyl-carrier protein] reductase	483,2	1.86	-	-
PAAG_02111	40S ribosomal protein S0	3130,3	1.55	-	-
PAAG_08955	40S ribosomal protein S1	672,2	1.62	-	0.720
PAAG_06367	40S ribosomal protein S11	3734,9	1.68	-	-
PAAG_08634	40S ribosomal protein S12	1542,5	1.80	-	-
PAAG_01413	40S ribosomal protein S17	1850,1	*	-	0.738
PAAG_09043	40S ribosomal protein S2	982,0	2.01	-	-
PAAG_03322	40S ribosomal protein S20	1303,5	1.63	-	0.750

PAAG_07847	40S ribosomal protein S26E	409,6	*	-	0.613
PAAG_02634	40S ribosomal protein S6-A	3851,9	1.65	-	-
PAAG_00468	4-aminobutyrate aminotransferase	977,2	1.75	-	0.601
PAAG_00724	60S ribosomal protein L11	2233,1	1.51	-	-
PAAG_00969	60S ribosomal protein L15	4374,1	1.60	-	-
PAAG_00430	60S ribosomal protein L2	360,1	1.55	-	0.853
PAAG_00952	60S ribosomal protein L20	1022,6	1.67	-	0.712
PAAG_01050	60S ribosomal protein L30	3630,4	1.67	-	-
PAAG_01178	6-phosphogluconate dehydrogenase. decarboxylating 1	1568,3	1.73	-	-
PAAG_07786	Acetyl-CoA acetyltransferase	1043,1	4.10	-	0.655
PAAG_03532	Actin	3976,5	1.70	-	-
PAAG_08620	ADP, ATP carrier protein	7031,9	1.63	-	-
PAAG_00403	Alcohol dehydrogenase 1	562,0	2.89	-	-
PAAG_05249	Aldehyde dehydrogenase	1357,1	1.86	-	-
PAAG_02603	Aspartate aminotransferase	303,7	1.92	-	-

PAAG_05117	Aspartate-tRNA(Asn) ligase	565,1	5.53	-	0.609
PAAG_04838	ATP synthase subunit 4, mitochondrial	663,4	1.51	-	0.781
PAAG_04820	ATP synthase subunit alpha, mitochondrial	1625,2	1.93	-	-
PAAG_04570	ATP synthase subunit D, mitochondrial	2347,4	1.54	-	-
PAAG_00689	ATP-dependent RNA helicase eIF4A	3109,0	1.92	-	-
PAAG_04511	ATP-dependent RNA helicase SUB2	2259,1	1.60	-	0.722
PAAG_03701	BAR domain-containing protein	844,8	2.48	-	0.614
PAAG_00731	Bifunctional purine biosynthesis protein ADE17	767,5	1.72	-	-
PAAG_05518	Cell division control protein 48	295,9	1.63	-	-
PAAG_00797	Chaperone DnaJ	1577,9	1.99	-	-
PAAG_01262	Chaperone DnaK	2982,3	1.57	0.864	-
PAAG_08075	Citrate synthase. mitochondrial	730,3	1.99	-	-
PAAG_08088	Cytochrome b-c1 complex subunit 2	603,4	1.54	-	-
PAAG_03292	Cytochrome c peroxidase, mitochondrial	3045,4	1.60	-	0.809
PAAG_06751	DNA damage checkpoint protein rad24	1203,8	2.05	-	-

PAAG_00173	Electron transfer flavoprotein subunit alpha	465,5	1.88	-	0.642
PAAG_03556	Elongation factor 1 gamma domain-containing protein	998,9	1.77	-	-
PAAG_11418	Elongation factor 1-alpha	14708,7	1.79	-	-
PAAG_00594	Elongation factor 2	3787,4	1.51	-	-
PAAG_00850	Glutamine-fructose-6-phosphate transaminase (isomerizing)	1305,8	1.65	-	0.693
PAAG_08093	GTP-binding protein ypt3	690,3	2.05	-	-
PAAG_03900	GTP-binding protein YPT52	440,3	*	-	-
PAAG_06811	Heat shock protein STI1	379,4	*	-	-
PAAG_07098	Histone H4.1	4254,4	1.62	-	-
PAAG_00126	Histone H4.2	4337,0	1.62	0.792	-
PAAG_08003	HSP72-like protein	4380,2	1.73	-	-
PAAG_07775	HSP75-like protein	472,9	2.10	-	-
PAAG_05679	HSP90-like protein	8089,5	1.68	-	-
PAAG_02130	HSP98-like protein	572,8	2.08	-	-

PAAG_00053	Malate dehydrogenase, NAD-dependent	1037,2	1.62	-	0.651
PAAG_02718	Mannose-1-phosphate guanyltransferase	427,5	2.18	-	-
PAAG_00481	Membrane biogenesis protein Yop1	922,3	1.93	-	0.902
PAAG_01861	Membrane-associated progesterone receptor component 1	443,0	*	-	0.735
PAAG_02265	Mitochondrial F1F0 ATP synthase subunit	1739,0	1.99	-	-
PAAG_12425	Mitochondrial outer membrane protein porin	1423,3	1.93	-	-
PAAG_06891	mRNA binding post-transcriptional regulator (Csx1)	1068,9	2.23	-	-
PAAG_12076	NAD(P)H:quinone oxidoreductase, type IV	1770,3	1.55	0.718	-
PAAG_00953	NADH-cytochrome b5 reductase 2	544,5	1.62	-	-
PAAG_05735	NADH-ubiquinone oxidoreductase 49 kDa subunit, mitochondrial	668,4	2.05	-	0.675
PAAG_08100	O-acetylhomoserine (thiol)-lyase	313,5	2.29	-	-
PAAG_01321	Oxidoreductase 2-nitropropane dioxygenase family	2254,9	1.79	-	0.707
PAAG_00739	Peptidyl-prolyl cis-trans isomerase B	583,7	1.65	0.641	-

PAAG_03334	Peptidyl-prolyl cis-trans isomerase D	4124,6	1.99	-	-
PAAG_01454	Peroxisomal catalase	2765,8	2.29	-	-
PAAG_08859	Peroxisomal multifunctional enzyme	460,3	1.80	-	-
PAAG_08082	Plasma membrane ATPase	738,9	1.93	-	0.712
PAAG_07957	Pre-mRNA splicing factor	539,9	2.69	-	0.801
PAAG_04458	Prohibitin-1	1883,9	1.62	-	-
PAAG_11504	Protein disulfide-isomerase domain	364,5	*	-	0.783
PAAG_00986	Protein disulfide-isomerase domain	585,2	1.90	-	-
PAAG_02050	Pyruvate decarboxylase	1078,4	1.84	-	-
PAAG_02769	Pyruvate dehydrogenase protein X component	325,5	*	-	0.685
PAAG_00417	Succinyl-CoA ligase subunit alpha	1400,3	1.88	-	0.624
PAAG_02921	Translation elongation factor Tu	990,0	1.70	-	0.773
PAAG_03031	Tubulin beta chain	1417,8	1.63	-	-
PAAG_04901	Ubiquitin-conjugating enzyme	555,9	1.79	-	0.883
PAAG_12424	Voltage-dependent anion channel protein 1	2827,7	1.54	-	0.761

[a]Protein accession number in NCBI, available at https://www.ncbi.nlm.nih.gov/protein.

[b]Description of the protein in the *Paracoccidioides* spp. databank, available in https://www.uniprot.org/uniprot/?query=paracoccidioides+lutzii&sort=score.
[c]Score that points to the quality of protein identification. Only proteins with satisfactory score values were included in the analysis.
[d]Ratio between quantification of proteins identified in hemoglobin/iron deprivation. Values ≥ 1.5 indicate upregulated proteins; (*) indicates that the protein was identified only upon hemoglobin treatment.
[e]Prediction of signal peptide presence; score must be ≥ 0.45; prediction performed by SignalP 4.1 available at http://www.cbs.dtu.dk/services/SignalP/. (-) indicates that the signal peptide was not identified.
[f]Prediction of protein secretion by non-classical pathways. whose score must be ≥ 0.6; prediction performed by SecretomeP 2.0 available at http://www.cbs.dtu.dk/services/SecretomeP/. (-) indicates that the protein was not predicted as secreted.

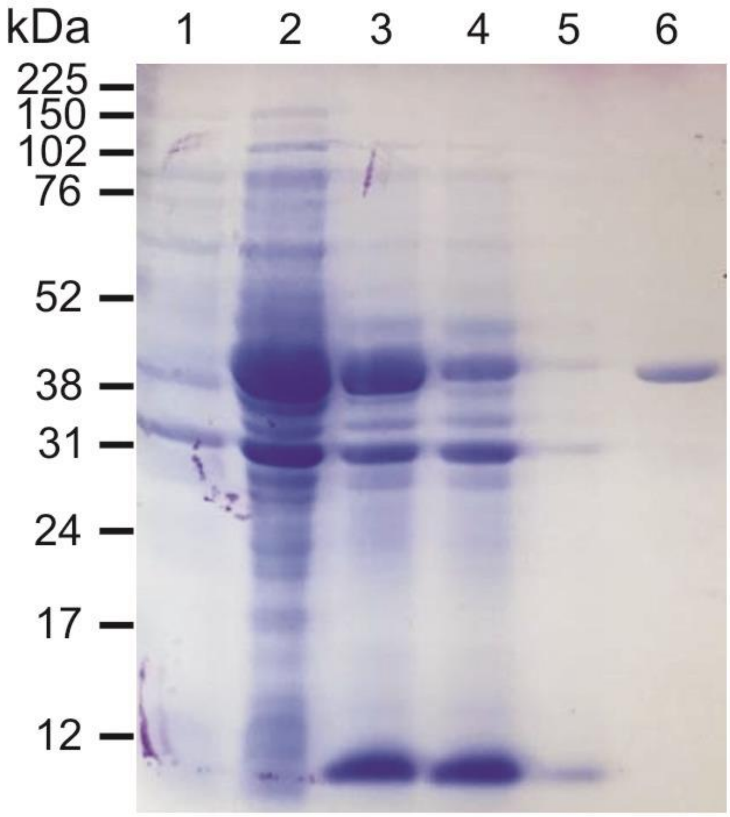

Supplementary Figure 2. Expression and purification of *Pb*HSP30. Heterologous expression of *Pb*HSP30. 1 – *E. coli* C43 bacteria harbouring the plasmid pET-32a containing the *HSP30* coding sequence without the addition of IPTG. 2 – Induction of HSP30 expression with IPTG 0.1 mM for 16 h in the bacteria *E. coli* C43 harbouring the plasmid pET-32a containing the *HSP30* coding sequence. 3 – Pellet of the protein extract from HSP30 induction after sarcosyl treatment. 4 – supernatants of the protein extract from HSP30 induction after sarcosyl treatment. 5 - nickel resin washing. 6 – purification of the HSP30 recombinant protein through nickel resin. The estimated molecular weight of the recombinant protein is 46 kDa.

Supplementary Table 3. Analysis of all-atom contacts and geometry of the HSP30-hemoglobin complex

**Protein Geometry**	**Before DM**	**Percentage**	**After DM**	**Percentage**
Poor rotamers	85	19.50%	14	2.13%
Favored rotamers	300	68.81%	600	91.19%
Ramachandran outliers	29	7.53%	10	1.25%
Ramachandran favored	293	76.10%	726	90.41%
Bad bonds	103	1.60%	0	0.00%
Twisted peptides	11	2.14%	5	0.62%

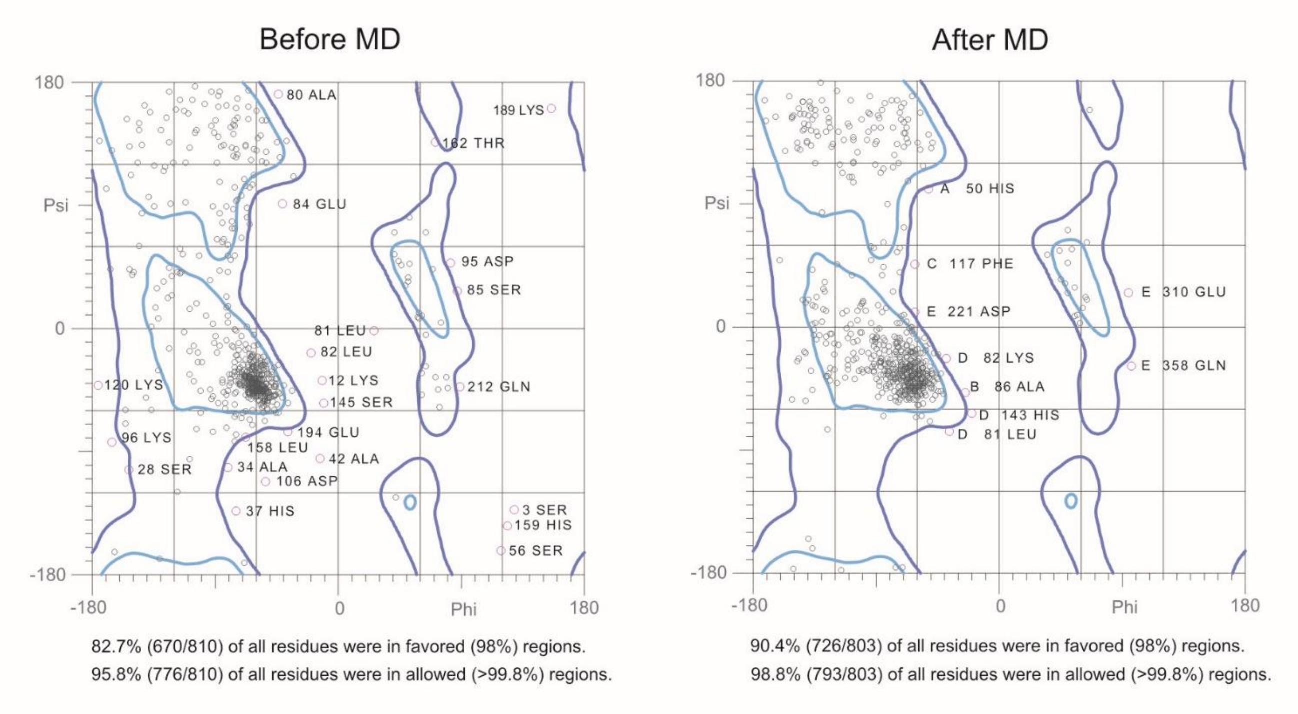

Supplementary Figure 3. Ramachandran plots evaluation. Ramachandran plots showing the analysis of residues in allowed, favored and forbidden regions (**A**) before and (**B**) after the HSP30-hemoglobin complex molecular dynamics. The light blue lines represent favorable regions, and the dark blue lines represent allowed regions. Regions outside blue lines represent forbidden regions and may characterize very flexible regions of either HSP30 or hemoglobin. The plots show that stereochemistry of the complex improved considerably after molecular dynamics.

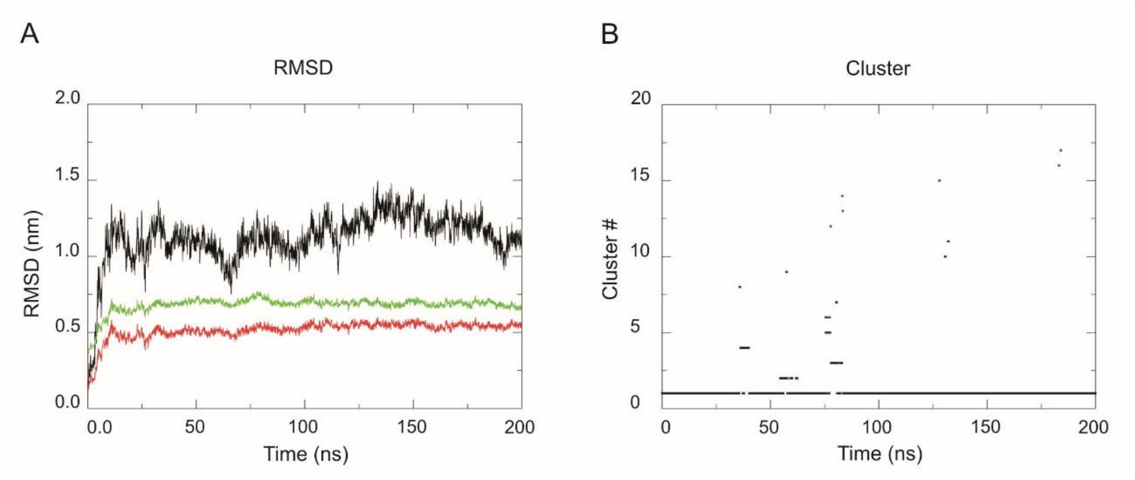

Supplementary Figure 4. Molecular Dynamics assessment. (**A**) RMSD (root mean square deviation) trajectories over 200 ns of the complex simulation. The complex formed by the interaction between HSP30 and hemoglobin achieved stability around 0.5 nm (green); hemoglobin achieved stability around 0.6 nm (red) and HSP30 conformation stabilized around 1.2 nm (black). The conformational structure of the complex is more stable than the proteins involved in the interaction taken individually. (**B**) The cluster plot shows eighteen sets of conformations along the HSP30-hemoglobin complex trajectories and the first cluster shows the most stable conformation persisting throughout the simulation.

A

```
P.lutzii HSP30             MFSRRAIRSPLKASRLPSQPTSSAYRISKFSSGAIPHIYPSATTTLSNINQARNMSFFPR
Human heme oxygenase1      MERPQPDSMPQDLSEALKEATKEVH--TQAENAEFMRNFQKGQVTRDGFKLVMASLYHIY
                           *   :.   * . *.  .:.*...:  :: ... : : : .. .* ..:: .    :.

P.lutzii HSP30             LPTGDFTPLFRLLDDYESHRSGVESSSHLGGMRTFAPRFDVREAKDAYHLDGELPGINQK
Human heme oxygenase1      VALEEEIERNKESPVFAPVYFPEELHRKAALEQDLAFWYGP-RWQEVIPYTPAMQRYVKR
                           :.  :     :    : .     *   : .  : :*  :.  . ::.      :    ::

P.lutzii HSP30             DIEIEFTDSQTLVIKGRSEREYTSSSNGEPGDKGQKKLHQPTVEDESSTSMTKTGGSSQE
Human heme oxygenase1      LHEVGRTEPELLVAH-----AYTRYLGDLSGGQVLKKIAQKALDLPSSG----EGLAFFT
                             *:  *:.: ** :      **   .. .*.:  **: * :::  **      *  :

P.lutzii HSP30             VGSVREGVKYWVSERS-VGEFSRTFSFPTRVDQNAVKASLKNGILSVIIPKMSAPTSKKI
Human heme oxygenase1      FPNIASATKFKQLYRSRMNSLEMTPAVRQRVIEEAKTAFLLNIQLFEELQELLTHDTKDQ
                           . .: ...*:    ** :..:. * :.  ** ::* .* * *  *   : :: :  :*.

P.lutzii HSP30             TIE--
Human heme oxygenase1      SPSRA
                           : .
```

B

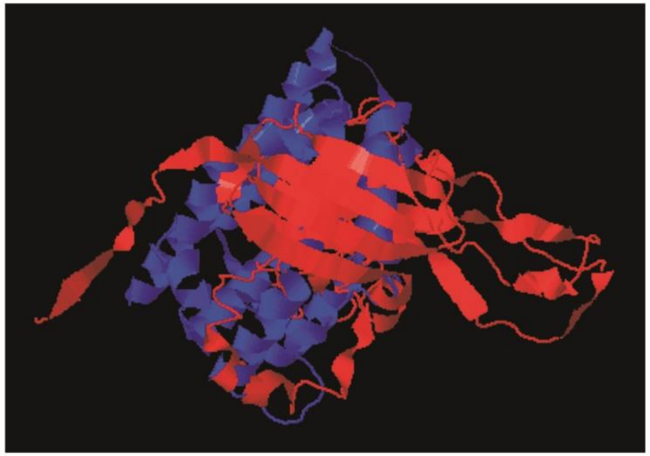

Supplementary Figure 5. Sequence and structural alignment of HSP30 (PAAG_00871) and heme oxygenase 1 (P09601). (**A**) The alignment was obtained by ClustalX 2.1 program. Asterisks (*) indicates position of complete identity, a colon (:) indicates conserved substitutions and a dot (.) indicates a semi-conserved substitution of amino acid residues. (**B**) Structural alignment was obtained by TM-align (https://zhanglab.ccmb.med.umich.edu/TM-align/). This algorithm is used to construct the best structural alignment between protein pairs according to the rotation of the alpha-carbons in amino acid residues.
